# Prokaryotic Communities at Different Depths between Soils with and without Tomato Bacterial Wilt but Pathogen-Present in a Single Greenhouse

**DOI:** 10.1264/jsme2.ME16136

**Published:** 2017-05-13

**Authors:** Chol Gyu Lee, Toshiya Iida, Yasuhiro Inoue, Yasunori Muramoto, Hideki Watanabe, Kazuhiro Nakaho, Moriya Ohkuma

**Affiliations:** 1 Japan Collection of Microorganisms, RIKEN BioResource CenterTsukuba, Ibaraki, 305–0074Japan; 2 Central Region Agricultural Research Center, National Agriculture and Food Research OrganizationTsukuba, Ibaraki, 305–8666Japan; 3 Gifu Prefectural Agricultural Technology Center729 Matamaru, Gifu 501–1152Japan

**Keywords:** 454 pyrosequencing, lower soil layer, prokaryotic diversity, tomato bacterial wilt

## Abstract

The characterization of microbial communities that promote or suppress soil-borne pathogens is important for controlling plant diseases. We compared prokaryotic communities in soil with or without the signs of tomato bacterial wilt caused by *Ralstonia solanacearum*. Soil samples were collected from a greenhouse at two different depths because this pathogen is present in deep soil. We used samples from sites in which we detected *phcA*, a key gene regulating *R. solanacearum* pathogenicity. The pyrosequencing of prokaryotic 16S rRNA sequences in four soil samples without disease symptoms but with *phcA* and in two soil samples with disease symptoms indicated that community richness was not significantly different between these two soils; however, microbial diversity in the lower soil layer was higher in soil samples without disease symptoms but with *phcA*. A difference in prokaryotic community structures between soil samples with and without bacterial wilt was only observed in the upper soil layer despite apparent similarities in the communities at the phylum level. *Proteobacteria*, *Acidobacteria*, *Chloroflexi*, *Verrucomicrobia*, and several Archaea were more abundant in soil samples without disease symptoms, whereas taxa in another eight phyla were more abundant in soil samples with disease symptoms. Furthermore, some prokaryotic taxa were abundant specifically in the lower layer of soil, regardless of whether disease was present. These prokaryotic taxa may suppress or accelerate the pathogenesis of bacterial wilt and are good targets for future studies on disease control.

Soil-borne pathogens cause various plant diseases such as take-all, damping off, crown rot, and wilting. Thus, pathogens pose a serious threat to crop production and food security. Bacterial wilt caused by the soil-borne pathogen *Ralstonia solanacearum* has been reported worldwide and is one of the most devastating plant diseases (26, 43). This pathogen infects more than 200 plant species, *e.g.*, olive, tomato, tobacco, and eggplant, and causes great losses in agriculture and horticulture (18). It is mainly distributed in soil and enters roots through injured tissue or natural openings. Several approaches to control bacterial wilt, including soil amendments, crop rotation, and field sanitation (39), have been suggested; however, a definitive control method has not yet been developed.

Three elements are generally needed for the onset of plant disease: a susceptible host, the presence of a pathogen, and a conducive environment. A conducive environment comprises appropriate temperatures, soil chemical properties, soil types, and other factors. One of the most important factors in the outbreak of a soil-borne pathogen is soil microbial properties. Previous studies compared bacterial communities between soil samples with and without soil-borne disease symptoms, and showed that specific bacteria are involved in pathogen suppression (3, 21, 23, 25, 33, 35). Comparisons of microbial communities between soil samples with and without soil-borne disease pathogens are important for the selection of effective pathogen-controlling microorganisms and a clearer understanding of interactions between soil microbial communities and soil-borne pathogens. High-throughput sequencing technologies such as 454 pyrosequencing have been used in several recent studies that compared bacterial communities between soil samples with and without soil-borne disease pathogens, including *Fusarium* wilt, black root rot, and *Rhizoctonia solani* (20, 32, 45). *R. solanacearum* is present in soil at depths greater than 40 cm, and causes disease in tomato plants because their roots reach into deep soil (11). Therefore, an analysis of prokaryotic communities in this deep layer of soil is important. However, to the best of our knowledge, microbial communities have not yet been examined in this soil layer. Furthermore, soil samples from different fields with or without plant disease have been analyzed (20, 32, 33, 45). Difficulties are associated with identifying disease-associated microorganisms using comparisons between soil samples from different fields because members of indigenous soil microbial communities may markedly vary between sampling sites.

In the present study, we compared prokaryotic communities in soil samples with and without plants presenting tomato bacterial wilt using high-throughput sequencing and identified possible prokaryotic taxa associated with the promotion or suppression of this disease. In order to achieve this, we collected soil samples from a single greenhouse and investigated soil samples at depths less than and greater than 40 cm because the latter is also relevant to bacterial wilt.

## Materials and Methods

### Soil sampling and DNA extraction

Soil samples were collected from a greenhouse of tomato plants located in Kaizu City, Gifu Prefecture (35°23′ N, 136°63′ E; Fluvaquentic Haplosaprists) in late January 2015. The locations of plots in this greenhouse are described in Supplemental Fig. S1. Tomato plants have been continuously cultivated without serious plant diseases for 3 and 26 years in the northern and southern parts of the greenhouse, respectively. Plants in some areas of the northern part of the greenhouse coincidentally showed bacterial wilt symptoms at the time of sampling, whereas plants in other parts of the greenhouse remained disease-free. Therefore, two plots with plants showing signs of disease (plots N1 and N2) and four plots with no plants showing signs of disease (plots N3 to N6) in the northern part, and six plots with no plants showing signs of disease in the southern part (plots S1 to S6) were collected using a core sampler (Gauge Auger DIK-106B, Daiki Rika Kogyo, Saitama, Japan). We also collected soil samples from two different depths (20–30 cm and 40–50 cm) in each plot. A total of 24 samples were collected and stored at −20°C until used. Average soil pH (H_2_O) were 7.04 and 7.02 and electric conductivities (EC) were 0.053 and 0.042 mS cm^−1^ in the upper and lower layers, respectively. Soil pH and EC in the same layer were not significantly different between the soil sites with and without bacterial wilt symptoms (*p*<0.05, *t*-test).

DNA was extracted from 0.5 g of soil with an ISOIL for the Beads Beating kit (Nippongene, Tokyo, Japan) following the manufacturer’s instructions. DNA was quantified and its integrity was measured using a NanoDrop spectrophotometer (Thermo Scientific, Waltham, MA, USA) and by visualization on a 0.8% agarose gel in Tris-acetate-EDTA (TAE) buffer. The amount of DNA extracted from each soil sample is presented in Supplemental Table S1.

### Detection of *R. solanacearum*

The *phcA* gene, which plays a major role in the regulation of *R. solanacearum* pathogenicity (31), was amplified from DNA extracted from each soil sample. A two-step nested polymerase chain reaction (PCR) was performed to detect *phcA* using two primer sets (16): phcA2981f (5′-TGGATATCGGGCTGGCAA-3′) and phcA4741r (5′-CGCTTTTGCGCAAAGGGA-3′) for the first step and phcA3538f (5′-GTGCCACAGCATGTTCAGG-3′) and phcA4209r (5′-CCTAAAGCGCTTGAGCTCG-3′) for the second step. PCR was performed in a total volume of 50 μL in a 200-μL microtube that contained 0.5 μL of KAPA2G Robust HotStart DNA Polymerase (5 U μL^−1^) (KAPA Biosystems, Wilmington, MA, USA), 5 μL of PCR reaction buffer for a high GC content (KAPA Biosystems), 1.5 μL of each primer (3.2 pmol each), 4 μL of a dNTP mixture (10 mM), 2.5 μL of MgCl_2_ (25 mM), 1 μL of a DNA template, and 35.5 μL Milli-Q water. The following PCR amplification profile was used: 95°C for 2 min, followed by 30 cycles of denaturation at 95°C for 30 s, annealing at 57.5°C for 30 s, and extension at 72°C for 60 s, with a final extension at 72°C for 5 min. One microliter of the PCR product from the first-step amplification was used as a template for the second-step PCR reaction. Second-step PCR conditions were the same as those in first-step PCR, except that the extension time was reduced to 30 s. Amplification was verified by gel electrophoresis (1.5% agarose in TAE buffer).

### Tag-encoded amplicon pyrosequencing

PCR was performed with each soil sample in order to amplify the V4 variable region of 16S rRNA using the bacterial and archaeal universal primers 515F and 806R (6) coupled with Roche 454 Titanium sequencing adapters. The 515F primer (5′-GTGCCAGCMGCCGCGGTAA-3′) contained a 10-base pair (bp) bar-coded sequence with a Roche 454-A pyrosequencing adapter (Titanium Lib-L adapters), and a Roche 454-B adapter for the 806R primer (5′-GGACTACVSGGGTATCTAA-3′). PCR was performed in a 200-μL microtube with a total volume of 50 μL, consisting of 0.5 μL of each primer (50 pmol each), 5 μL of a 2.5 mM dNTP mixture, 5 μL of 10×Ex Taq DNA buffer (20 mM Mg^2+^; TaKaRa, Otsu, Japan), 0.25 μL of Ex Taq DNA polymerase (5 U μL^−1^) (TaKaRa), 1 μL of a DNA template, and 37.75 μL of Milli-Q water. The following PCR amplification profile was used: an initial denaturation at 94°C for 3 min, followed by 30 cycles of 94°C for 45 s, 50°C for 30 s, and 72°C for 90 s, and a final extension step of 72°C for 10 min. In 1.5% agarose gels showing two or more bands, PCR products of approximately 350 bp in length were excised from the agarose gel and purified using a MonoFas DNA purification kit (GL Sciences, Tokyo, Japan). Each PCR amplicon was cleaned twice using an Agencourt AMPure XP system (Beckman Coulter, Pasadena, CA, USA) to remove primers and short DNA fragments, and then quantified using a Qubit Fluorometer (Invitrogen, Carlsbad, CA, USA). The purified PCR amplicons were combined in equimolar ratios into a single tube for emulsion PCR (emPCR). An emPCR reaction was performed with an approximate ratio of 0.2:1 (amplicon:emPCR beads), and amplicon sequencing was performed following the manufacturer’s protocols (Roche Applied Science, Indianapolis, IN, USA) on a Roche 454 GS Junior Titanium sequencer using a Lib-L kit. Sequencing data were deposited in the DNA Data Bank of Japan (DDBJ) Sequence Read Archive under accession number DRA004754.

### Data analysis

Raw standard flowgram format files were pre-processed in Quantitative Insights Into Microbial Ecology (QIIME) (5). Data from read sequences, quality, flows, and ancillary metadata were analyzed using the QIIME pipeline according to Campisaono *et al.* (4). Quality filtering consisted of discarding reads of <200 bp or >1,000 bp in length, excluding homopolymer runs of >6 bp and continuous ambiguous bases of >6, accepting one barcode correction and two primer mismatches. Moreover, reads with mean quality scores less than 25 were also removed. Singleton operational taxonomic units (OTUs) and chimeric sequences were then removed before the statistical analysis. Denoising was performed using the built-in Denoiser algorithm, and chimera removal and OTU picking were accomplished with USEARCH 61, based on pairwise identities of 0.97. Taxonomies were assigned with the Ribosomal Database Project (RDP) naïve Bayesian classifier with a minimum confidence of 0.8 against the Greengenes Database (October 2012 release). An OTU-based analysis was performed with pyrotag-based datasets to calculate richness and diversity using the phyloseq package in R (1.7.24) (24). The diversity within each individual sample was estimated using non-parametric Shannon and Simpson diversity indices. The Chao1 estimator and abundance-based coverage estimator (ACE) were calculated to estimate the richness of each sample. We used the Student’s *t*-test to assess differences in prokaryotic diversity and richness (*p*<0.05). A multivariate analysis of community structure and diversity were performed with pyrotag-based datasets using a weighted UniFrac dissimilarity matrix calculated in QIIME, jackknifing (1,000 reiterations), read abundance data at the deepest level possible (3,105 reads), and unconstrained ordination in a principal coordinate analysis (PCoA). Indicator values were then calculated using the indicspecies package in R (8), with the aim of identifying OTUs associated with soils without disease symptoms rather than soils with disease symptoms or vice versa (*p*<0.05). In order to calculate indicator values, 999 random permutation tests were performed.

## Results and Discussion

### Detection of pathogenic *R. solanacearum*

The presence of *R. solanacearum* in each soil sample was assessed by the detection of *phcA*, which plays a major role in the regulation of pathogenicity (31). The PCR products of *phcA* were obtained from all sites with disease, irrespective of soil depth. *phcA* was detected in four out of 10 sites without plant wilt disease (Table 1, Supplemental Fig. S2). In these four sites, pathologies associated with *R. solanacearum* were not observed. We classified soil samples into three types (Table 1): soil not showing the signs of disease but with *phcA* (ND-soil), soil with diseased tomato plants and *phcA* (D-soil), and soil not showing bacterial wilt or *phcA* (H-soil). We selected 4 ND-soil samples and 2 D-soil samples for subsequent experiments. The H-soil sites in this field were not included because we were unable to evaluate microbial communities for disease without the presence of the pathogen. Amplicons of *phcA* were detected in the lower layer (40–50 cm depth) of soil in all D-soil and ND-soil sites, except for one (plot S2), indicating that *R. solanacearum* was present in soil at a depth greater than 40 cm, as described in a previous study (11).

In some soil environments, the incidence of the disease is known to be reduced in spite of the presence of pathogens, susceptible host plants, and climatic conditions favorable for disease development (2, 12, 13, 19). This soil, called disease-suppressive soil, has been reported for multiple soil-borne pathogens, including those causing *Fusarium* wilt (22), potato common scab (17), damping-off disease (15), tobacco black root rot (20), and bacterial wilt (33). However, in the present study, ND-soil with *phcA* was not defined as suppressive soil because we did not experimentally confirm its suppressive effects on bacterial wilt.

### Richness, diversity, and microbial community structure

Pyrosequencing yielded a total of 105,967 high-quality sequences from the 12 samples. These sequences were clustered into 18,449 OTUs. A total of 1,452 and 1,206 OTUs were observed in the upper layers of ND- and D-soil, respectively, whereas 1,222 and 1,111 OTUs, respectively, were detected in the lower layers (Table 2). The number of OTUs and Chao1 and ACE richness indices were not significantly different between ND- and D-soil. However, the Shannon and Simpson diversity indices were significantly higher in D-than in ND-soil in the lower layer (each *p*<0.05). Previous studies indicated that the compositions of indigenous bacterial populations are simpler in conducive soil than in suppressive soil (32, 33). Therefore, some unique microbes may be involved in promoting or suppressing bacterial wilt disease.

DNA concentrations in the upper layer of soil were approximately three-to seven-fold higher than those in the lower layer (*p*<0.05) (Supplemental Table S1). Shannon and Simpson diversity indices were also higher in the upper layer (*p*<0.05). Watanabe *et al.* (40) indicated that the total DNA concentration in bulk soil was markedly reduced at depths greater than 30 cm because of the low carbon and nitrogen contents. The field in this study was plowed to a depth of 30 cm, and the use of an agrimotor may have consolidated the lower layer of soil. Thus, soil physicochemical properties may have differed between the upper and lower layers, and prokaryotes in the lower soil layer had a lower total microbial biomass and were less diverse than those in the upper layer (Table 2 and Supplemental Table S1).

**Fig. 1:**
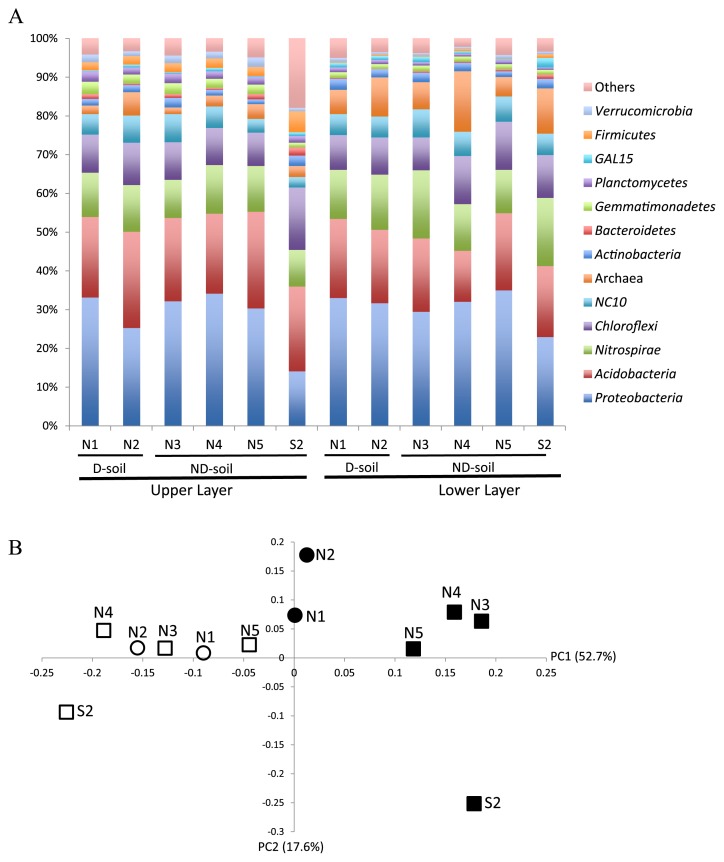
Relative abundances of prokaryotic phyla (A) and a UniFrac-weighted principal component analysis of prokaryotic communities in D- and ND-soil (B). (B) Closed circle: D-soil, upper layer. Open circle: D-soil, lower layer. Closed square: ND-soil, upper layer. Open square: ND-soil, lower layer.

A total of 54 phyla were detected in the 12 soil samples. *Proteobacteria* and *Acidobacteria* accounted for 36% to 55% of the prokaryotic OTUs in all samples (Fig. 1A). Furthermore, *Acidobacteria*, *Gemmatimonadetes*, *Planctomycetes*, and *Firmicutes* were significantly more abundant in the upper layer than the lower layer, whereas *Nitrospirae* was less abundant (each *p*<0.05) (Fig. 1A). However, the relative abundance of each prokaryotic phylum was not significantly different between ND- and D-soil. These results indicate that prokaryotic community compositions were not significantly different at the phylum level between soil with and without disease.

A weighted UniFrac analysis and PCoA showed that the prokaryotic communities in soil samples were classifiable into two groups according to soil depth (Fig. 1B). The community structures in the upper soil layer were also distinct between ND- and D-soil, whereas those in the lower soil layer were similar (Fig. 1B). Therefore, a difference in prokaryotic community structures between ND- and D-soil was only observed in the upper layer of soil collected from a single greenhouse; however, this difference was not apparent at the bacterial phylum level. The prokaryotic communities of soil sample S2, collected from the southern part of the field, were distantly related to samples collected from the northern part, both in the upper and lower layers, suggesting that the prokaryotic community structure differed according to soil management practices rather than the observation of bacterial wilt.

### Specific OTUs in ND-soil

Twenty-five OTUs were unique or significantly more abundant in ND-soil than in D-soil in their respective layers (Table 3). Among them, seven OTUs were commonly detected in the upper and lower layers, whereas eight and 10 OTUs were detected in one of the layers only. OTUs belonged to the following phyla: *Acidobacteria* (8 OTUs), *Proteobacteria* (8 OTUs), *Chloroflexi* (4 OTUs), *Aenigmarchaeota* (1 OTU), *Euryarchaeota* (1 OTU), *Crenarchaeota* (1 OTU), *Woesearchaeota* (1 OTU), and *Verrucomicrobia* (1 OTU).

*Acidobacteria* was predominant in ND-soil. *Acidobacteria* subdivisions 1, 3, 4, and 6 are the most abundant bacteria in agricultural soil (17). In this study, OTUs belonging to subdivisions 1, 2, 3, 4, 12, and 13 were specifically abundant in ND-soil. Previous studies showed that *Acidobacteria* subdivisions 4 and 6 were more abundant in soil that suppresses potato common scab and *Fusarium* wilt (29, 32). Moreover, the abundance of *Acidobacteria* subdivisions 1 and 3 negatively correlated with that of *R. solanacearum* (41). However, previous studies also demonstrated that some members of this phylum are more abundant in soil with plants showing signs of disease due to several soil-borne pathogens (21, 30, 32, 41). These bacteria may play a role in preventing bacterial wilt; however, the ecology of *Acidobacteria* has not yet been defined in detail.

The proteobacterial genera *Hephaestia*, *Cystobacterineae*, *Azospirillum*, *Nitrosospira*, *Denitratisoma*, *Desulfuromonas*, *Pseudoduganella*, and *Dyella* were abundant in ND-soil. *Azospirillum* and *Dyella* were previously detected in disease-suppressive soil (4, 20). The detection of *Azospirillum* in ND-soil is interesting because they are known as plant growth-promoting bacteria and for their nitrogen-fixing ability (34, 46). *Nitrosospira*, *Denitratisoma*, and *Dyella* are also involved in the nitrogen cycle in soil environments (9, 36, 37). The suppressive effect on plant disease has been discussed for bacteria that participate in the nitrogen cycle and the nitrogen they release has been found to affect microbial community structures in soil (7, 14). Therefore, bacteria that play a role in the nitrogen cycle in soil may be involved in the control of bacterial wilt.

*Anaerolineae*, belonging to *Chloroflexi*, were also abundant in ND-soil. These bacteria are neutrophilic, strictly anaerobic chemo-organotrophs that have the ability to utilize sugars and polysaccharides (28). They inhabit extremely diverse environments, including sediments, subsurface habitats, anaerobic-dechlorinating environments, hot springs, deep hot aquifers, and anaerobic wastewater sludges (44). They have relatively long doubling times (45–100 h) in cultures; therefore, they are difficult to isolate. We detected not only bacteria, but also Archaea in the phyla *Aenigmarchaeota*, *Euryarchaeota*, *Crenarchaeota*, and *Woesearchaeota* in ND-soil. To the best of our knowledge, we are the first to have detected Archaea in suppressive soil. We are also the first to have detected and analyzed *Chloroflexi* and Archaea in ND-soil associated with tomato bacterial wilt using pyrosequencing methods.

*Woesearchaeota*, *Euryarchaeota*, *Aenigmarchaeota*, *Acidobacteria* subdivision 12 and 13, *Desulfuromonadales*, and *Azospirillum* were specifically detected in the lower layer of ND-soil. These bacteria are found in oligotrophic and anaerobic environments (27, 38). Due to these conditions, which are specific to the lower layer of soil, unique microbes were detected in the lower soil layer. Moreover, prokaryotes were less diverse in ND-than in D-soil (Table 2). Therefore, these indicator bacteria may have important roles in the suppression of bacterial wilt because tomato roots are found in deep (<40 cm) soil.

### Specific OTUs in D-soil

In D-soil, 37 indicator OTUs belonging to eight phyla were detected as significantly abundant OTUs (Table 4). OTUs belonging to *Actinobacteria* and *Planctomycetes* were specifically detected in D-soil rather than in ND-soil. The OTUs of *Acidobacteria* (7 OTUs) were dominant in the upper layer, whereas those of *Proteobacteria* (6 OTUs) and *Acidobacteria* (6 OTUs) were mainly detected in the lower layer of D-soil. We specifically detected OTUs belonging to *Acidobacteria* subdivisions 7 and 25 and *Holophaga* subdivision 8 in D-soil. Moreover, unique OTUs belonging to subdivisions 2, 3, 12, and 13 were found in ND- and D-soil. Members of *Acidobacteria*, *Proteobacteria*, and *Actinobacteria* were more abundant in soil with the appearance of disease (20, 21, 32). OTUs belonging to *Planctomycetes* were more abundant in D-soil, which is in contrast to previous findings indicating that they are frequency observed in soil and are aerobic heterotrophs (1, 22, 30, 42). Moreover, *Planctomycetes* bacteria have a unique metabolism and the ability to oxidize ammonia under anaerobic conditions (10). The abundance of these bacteria correlated with the appearance of disease; therefore, they may be associated with bacterial wilt.

## Conclusions

In the present study, we compared prokaryotic communities in upper and lower layers of soil with or without bacterial wilt using a 454 pyrosequencing analysis. We classified ND- and D-soil according to the appearance of a plant pathogen in the field depending on the detection of *phcA* in both soil types. In the lower layer of soil, prokaryotic diversity was less in ND-than in D-soil. A difference in prokaryotic community structures between ND- and D-soil was only observed in the upper layer of soil, despite an apparent similarity at the phylum level, and this may have been because soil was sampled from a single greenhouse. Twenty-five and 37 indicator OTUs were found to be more abundant in ND- and D-soil, respectively. Some bacteria and Archaea were specifically detected in the lower layer of ND-soil. The specific microbes detected in ND-soil may play important roles in suppressing the soilborne pathogens of bacterial wilt, and future studies are needed in order to clarify the roles of the prokaryotes detected.

## Supplementary material



## Figures and Tables

**Table 1:** Signs of disease and *phcA* gene amplification from each soil plot examined in this study.

Sampling field	Plot number	Soil layer	Disease	*phcA* gene	Soil type
Northern part	N1	Upper	+	+	D-soil
		Lower	+	+	
	N2	Upper	+	+	D-soil
		Lower	+	+	
	N3	Upper	−	−	ND-soil
		Lower	−	+	
	N4	Upper	−	−	ND-soil
		Lower	−	+	
	N5	Upper	−	+	ND-soil
		Lower	−	+	
	N6	Upper	−	−	H-soil
		Lower	−	−	

Southern part	S1	Upper	−	−	H-soil
		Lower	−	−	
	S2	Upper	−	+	ND-soil
		Lower	−	−	
	S3	Upper	−	−	H-soil
		Lower	−	−	
	S4	Upper	−	−	H-soil
		Lower	−	−	
	S5	Upper	−	−	H-soil
		Lower	−	−	
	S6	Upper	−	−	H-soil
		Lower	−	−	

ND-soil indicates soil without disease but with *phcA* present; D-soil indicates soil with disease and *phcA*; and H-soil indicates soil without bacterial wilt or *phcA*.

**Table 2:** Diversity and richness indices of prokaryotes in D- and ND-soil.

	Upper layer		Lower layer	
		
	D-soil	ND-soil	D-soil	ND-soil
Observed OTUs	1452±136	1206±364	1222±150	1111±243
Chao1	3609±736	3109±1306	3226±249	2994±743
ACE	3989±837	3254±1429	3323±129	3256±828
Shannon	4.35±0.17	4.46±0.08	4.15±0.14	4.01±0.13*
Simpson	0.973±0.004	0.976±0.002	0.970±0.004	0.965±0.007*

Asterisks represent pairs of means that are significantly different between D-soil (*n*=2) and ND-soil (*n*=4) in each layer (*p*<0.05).

**Table 3:** Operational taxonomic units (OTUs) abundant in ND-soil.

Soil layer	OTU number	Phylum	Closest relative	D-soil	ND-soil	D-soil	ND-soil
	
				Upper layer		Lower layer	
Upper layer	OTU239890	*Acidobacteria*	*Acidobacteria* Gp2	0	2.53 E-04	0	0
	OTU17668	*Acidobacteria*	*Acidobacteria* Gp3	4.76 E-04	2.43 E-03	4.82 E-05	3.09 E-05
	OTU4234	*Chloroflexi*	*Anaerolinae*	0	6.32 E-05	0	0
	OTU4724	*Chloroflexi*	*Anaerolinae*	1.43 E-04	8.22 E-04	0	0
	OTU723819	*Crenarchaeota*	*Thermoportei*	4.76 E-05	2.43 E-03	4.82 E-05	3.09 E-05
	OTU952203	*Proteobacteria*	*Pseudodugonella* sp.	9.52 E-05	4.11 E-04	0	0
	OTU21359	*Proteobacteria*	Cystobacterineae	0	1.90 E-04	0	0
	OTU2761428	*Proteobacteria*	*Dyella*	0	2.85 E-04	0	0

Lower layer	OTU8713	*Acidobacteria*	‘*Candidatus* Koribacter’	4.76 E-04	0	0	3.09 E-04
	OTU14103	*Acidobacteria*	*Acidobacteria* Gp12	0	0	0	2.47 E-04
	OTU4584	*Acidobacteria*	*Acidobacteria* Gp13	0	0	9.64 E-05	2.47 E-04
	OTU1107276	*Acidobacteria*	*Acidobacteria* Gp2	4.76 E-04	0	4.82 E-05	2.17 E-04
	OTU18194	*Aenigmarchaeota*	‘*Candidatus* Aenigmarchaeum’	4.76 E-04	0	0	2.47 E-04
	OTU19114	*Chloroflexi*	*Anaerolineaceae*	0	0	4.82 E-05	1.24 E-04
	OTU889	E*uryarchaeota*	*Methanomassiliicoccus*	0	0	9.64 E-05	4.33 E-04
	OTU16969	*Proteobacteria*	*Azospirillum* sp. EP3-3L	4.76 E-04	0	0	3.09 E-04
	OTU14184	*Proteobacteria*	*Desulfurmonadales*	0	0	1.45 E-04	4.64 E-04
	OTU21385	*Woesearchaeota*	*Woesearchaeota* Incertae Sedis AR15	4.76 E-04	0	0	1.86 E-04

Upper and lower layers	OTU2240	*Acidobacteria*	*Acidobacteria* Gp1	0	2.40 E-03	0	6.19 E-05
	OTU663880	*Acidobacteria*	*Acidobacteria* Gp4	0	1.90 E-04	9.64 E-05	9.90 E-04
	OTU23191	*Chloroflexi*	*Anaerolineaceae*	0	6.32 E-05	9.64 E-05	3.71 E-04
	OTU142261	*Proteobacteria*	*Hephaestia* sp.	4.76 E-04	7.27 E-04	0	6.19 E-05
	OTU10519	*Proteobacteria*	*Nitrosospira* sp. APG3	0	2.85 E-04	0	3.09 E-05
	OTU21362	*Proteobacteria*	*Denitratisoma* sp.	0	5.38 E-04	0	2.47 E-04
	OTU2781	*Verrucomicrobia*	*Spartobacteria* sp.	0	5.38 E-04	0	2.47 E-04

The relative abundances of OTUs are shown in the table. Each OTU was identified using an indicspecies analysis (*p*<0.05).

**Table 4:** Operational taxonomic units (OTUs) abundant in D-soil.

Soil layer	OTU number	Phylum	Closest relative	D-soil	ND-soil	D-soil	ND-soil
	
				Upper layer		Lower layer	
Upper layer	OTU15038	*Acidobacteria*	‘*Candidatus* Koribacter’	2.4E-04	0	0	0
	OTU17589	*Acidobacteria*	*Acidobacteria*_Gp13	1.4E-04	0	0	0
	OTU16659	*Acidobacteria*	*Acidobacteria*_Gp2	1.9E-04	0	0	0
	OTU4398	*Acidobacteria*	*Acidobacteria*_Gp2	2.9E-04	3.2E-05	0	0
	OTU16427	*Acidobacteria*	*Acidobacteria*_Gp3	1.4E-04	0	0	0
	OTU1216	*Acidobacteria*	*Acidobacteria*_Gp7	2.4E-04	6.3E-05	0	0
	OTU14433	*Acidobacteria*	*Acidobacteria*_Gp7	4.3E-04	3.2E-05	0	6.2E-05
	OTU10268	*Planctomycetes*	*Pirellula*	2.4E-04	0	0	0
	OTU7125	*Planctomycetes*	*Planctomycetaceae*	3.3E-04	3.2E-05	0	0
	OTU9710	*Proteobacteria*	*Alphaproteobacteria*	9.5E-05	0	1.1E-03	1.5E-04
	OTU21086	*Proteobacteria*	*Betaproteobacteria*	1.4E-04	3.2E-05	0	0
	OTU1323	*Verrucomicrobia*	*Spartobacteria*	9.5E-05	0	0	0
	OTU17053	*Verrucomicrobia*	*Spartobacteria*_genera_incertae_sedis	2.9E-04	0	0	3.1E-05

Lower layer	OTU18877	*Acidobacteria*	*Acidobacteria*_Gp12	0	0	4.3E-04	6.2E-05
	OTU1053	*Acidobacteria*	*Acidobacteria*_Gp13	0	0	1.9E-04	3.1E-05
	OTU2699	*Acidobacteria*	Acidobacteria_Gp25	0	0	6.7E-04	1.2E-04
	OTU15771	*Acidobacteria*	*Acidobacteria*_Gp3	0	0	2.4E-04	0
	OTU7061	*Acidobacteria*	*Acidobacteria*_Gp3	0	6.3E-05	2.4E-04	0
	OTU8173	*Acidobacteria*	*Holophaga*	0	0	2.4E-04	6.2E-05
	OTU5670	*Actinobacteria*	*Actinobacteria*	0	0	9.6E-05	0
	OTU2126	*Actinobacteria*	*Actinobacteria*	0	0	6.7E-04	9.3E-05
	OTU21537	*Pacearchaeota*	*Pacearchaeota* Incertae Sedis AR13	0	0	9.6E-05	0
	OTU2970	*Woesearchaeota*	Unclassified*Woesearchaeota*	0	0	6.7E-04	1.5E-04
	OTU15020	*Chloroflexi*	*Anaerolineaceae*	0	0	9.6E-05	0
	OTU24535	*Proteobacteria*	*Rhodospirillales*	0	3.2E-05	2.9E-04	3.1E-05
	OTU80	*Proteobacteria*	*Deltaproteobacteria*	0	0	1.4E-04	0
	OTU6774	*Proteobacteria*	*Geobacter*	0	0	1.9E-04	0
	OTU3473	*Proteobacteria*	*Nannocystineae*	0	0	2.4E-04	0
	OTU23743	*Proteobacteria*	*Methylococcaceae*	0	0	1.9E-04	0
	OTU9686	*Proteobacteria*	*Gammaproteobacteria*	5.2E-04	1.3E-04	3.9E-04	0

Upper and lower layers	OTU15704	*Acidobacteria*	*Acidobacteria*_Gp25	9.5E-05	0	9.6E-05	0
	OTU9423	*Armatimonadetes*	*Chthonomonas*	4.8E-05	3.2E-05	1.9E-04	0
	OTU19666	*Chloroflexi*	*Anaerolineaceae*	1.4E-04	0	1.4E-04	3.1E-05
	OTU15429	*Planctomycetes*	‘*Candidatus* Kuenenia’	4.8E-05	0	4.8E-04	0
	OTU19754	*Proteobacteria*	*Betaproteobacteria*	5.7E-03	4.7E-04	1.1E-02	1.9E-03
	OTU6110	*Proteobacteria*	*Deltaproteobacteria*	5.2E-04	1.9E-04	5.8E-04	1.2E-04
	OTU377	*Proteobacteria*	*Gammaproteobacteria*	2.4E-04	3.2E-05	9.6E-05	3.1E-05

The relative abundances of OTUs are shown in the table. Each OTU was identified using an indicspecies analysis (*p*<0.05).
