# Supplementary material for: Prokaryotic Communities at Different Depths between Soils with and without Tomato Bacterial Wilt but Pathogen-Present in a Single Greenhouse
Source: Microbes Environ. 2017 May 13;32(2):118–24. doi: 10.1264/jsme2.ME16136 (PMC5478534; doi:10.1264/jsme2.ME16136)
Supplement: Supplementary file 1 [file 32_118_s1.pdf]

Sample number	Soil layer	DNA concentration ($\mu g\ g^{-1}$ dry soil)	Read number	Observed OTUs	Chao1	ACE	Shannon	Simpson	Soil type
N1	Upper	145	11394	2103	4129	4530	4.47	0.975	D-soil
	Lower	24	12240	1778	3402	3414	4.25	0.973	
N2	Upper	67	9548	1619	3088	3345	4.23	0.970	
	Lower	22	8979	1416	3050	3232	4.05	0.968	
N3	Upper	92	10051	2178	4713	5050	4.52	0.975	ND-soil
	Lower	29	10465	1805	3742	4108	4.06	0.965	
N4	Upper	157	9532	1769	3640	3755	4.42	0.974	
	Lower	20	8930	1516	3452	3744	4.01	0.969	
N5	Upper	185	3105	995	2130	2171	4.53	0.978	
	Lower	41	3475	952	2107	2275	4.13	0.971	
S2	Upper	29	8837	1130	1954	2039	4.37	0.978	
	Lower	10	9411	1238	2676	2895	3.84	0.955	

Supplemental Table S1. Prokaryotic diversity and richness in each soil sample

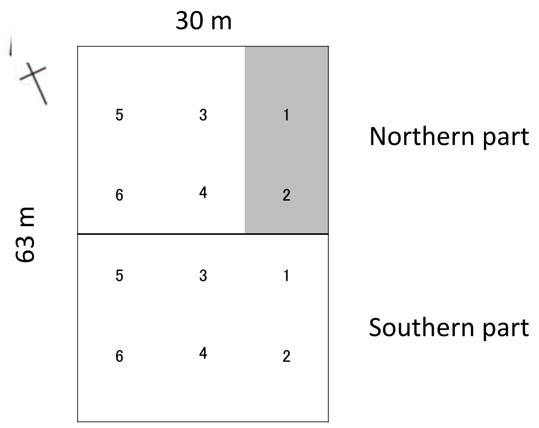

Supplemental Figure S1. Sample collection plots in the greenhouse

The sites with tomatoes showing signs of bacterial wilt are indicated in gray shading. Tomato plants were cultivated for 3 and 26 years in the northern and southern parts of the greenhouse, respectively. The greenhouse had been a paddy field before cultivation of tomato plants began. Soil solarization was treated before every planting.

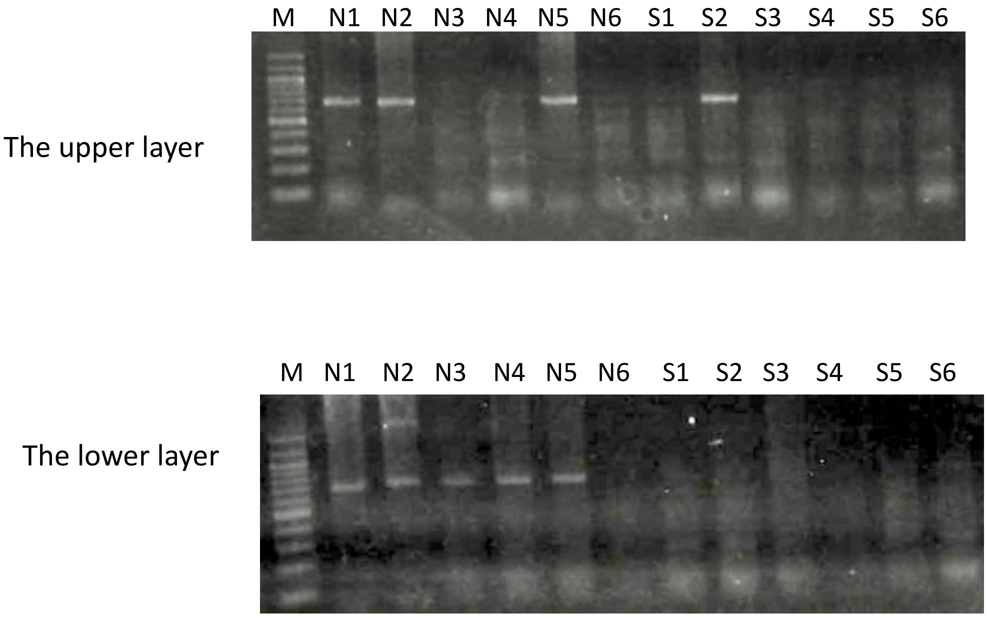

Supplemental Figure S2. PCR products of *phcA* in DNA extracted from each soil

M: 100-kb DNA ladder marker (Invitrogen, California, USA); N 1-6 and S 1-6 are the numbers of samples collected in the northern and southern parts of the field, respectively.
